# Automatic cortical representation of auditory pitch changes in Rett syndrome

**DOI:** 10.1186/s11689-016-9166-5

**Published:** 2016-09-01

**Authors:** John J. Foxe, Kelly M. Burke, Gizely N. Andrade, Aleksandra Djukic, Hans-Peter Frey, Sophie Molholm

**Affiliations:** 1 Department of Neuroscience, The Del Monte Institute for Neuroscience, University of Rochester School of Medicine and Dentistry, Rochester, NY 14642 USA; 2 Department of Pediatrics, The Sheryl and Daniel R. Tishman Cognitive Neurophysiology Laboratory, Albert Einstein College of Medicine & Montefiore Medical Center, Bronx, NY 10461 USA; 3 The Dominic P. Purpura Department of Neuroscience, Albert Einstein College of Medicine, Bronx, NY 10461 USA; 4 Department of Neurology, Rett Syndrome Center, Montefiore Medical Center & Albert Einstein College of Medicine, Bronx, NY 10467 USA; 5 Department of Neurology, Columbia University Medical Center, New York, NY 10032 USA

**Keywords:** High-density electrical mapping, EEG, Event-related potential, ERP, Auditory evoked potential, AEP, Mismatch negativity, MMN, MECP2, Females

## Abstract

**Background:**

Over the typical course of Rett syndrome, initial language and communication abilities deteriorate dramatically between the ages of 1 and 4 years, and a majority of these children go on to lose all oral communication abilities. It becomes extremely difficult for clinicians and caretakers to accurately assess the level of preserved auditory functioning in these children, an issue of obvious clinical import. Non-invasive electrophysiological techniques allow for the interrogation of auditory cortical processing without the need for overt behavioral responses. In particular, the mismatch negativity (MMN) component of the auditory evoked potential (AEP) provides an excellent and robust dependent measure of change detection and auditory sensory memory. Here, we asked whether females with Rett syndrome would produce the MMN to occasional changes in pitch in a regularly occurring stream of auditory tones.

**Methods:**

Fourteen girls with genetically confirmed Rett syndrome and 22 age-matched neurotypical controls participated (ages 3.9–21.1 years). High-density electrophysiological recordings from 64 scalp electrodes were made while participants passively listened to a regularly occurring stream of 503-Hz auditory tone pips that was occasionally (15 % of presentations) interrupted by a higher-pitched deviant tone of 996 Hz. The MMN was derived by subtracting the AEP to these deviants from the AEP produced to the standard.

**Results:**

Despite clearly anomalous morphology and latency of the AEP to simple pure-tone inputs in Rett syndrome, the MMN response was evident in both neurotypicals and Rett patients. However, we found that the pitch-evoked MMN was both delayed and protracted in duration in Rett, pointing to slowing of auditory responsiveness.

**Conclusions:**

The presence of the MMN in Rett patients suggests preserved abilities to process pitch changes in auditory sensory memory. This work represents a beginning step in an effort to comprehensively map the extent of auditory cortical functioning in Rett syndrome. These easily obtained objective brain measures of auditory processing have promise as biomarkers against which future therapeutic efforts can be assayed.

## Background

Given the severe clinical expression of classic Rett syndrome, where “methyl CpG binding protein 2” (*MECP2*) mutations lead to a precipitous regression phase that steals away motor skills and the ability to speak, we are left with only rudimentary understanding of the perceptual and cognitive capabilities of these children as they progress through childhood. While monitoring of eye-gaze patterns has proven a fruitful avenue of pursuit and has provided significant insights into preserved abilities [1–7], limitations of the approach are that it does not provide information regarding the integrity of underlying neural mechanisms and that it is highly reliant on the behavioral state and active participation of the patient. In this regard, non-invasive brain mapping techniques such as high-density electrophysiology allow for objective measures of brain function, provide insight into underlying cortical network dynamics, and allow for an assessment of the processing level at which information flow may be breaking down [8]. The event-related potential (ERP) technique provides researchers and clinicians with an exquisite tool to assess the initial sensory registration of a given input, the subsequent perceptual processing of that input, and in turn, the degree to which higher-order cognitive processing is engaged. Thus, ERPs can provide insight into mechanism and furnish us with objective biomarkers of neuropathology. As Byiers and Symons [9] have written, “The current lack of appropriate psycho-social assessments for this population makes outcome measurement in current and future clinical trials very challenging, and the need for unbiased, standardized assessments is urgent.”

It is perhaps surprising therefore that the evoked potential technique, so widely utilized in other neurodevelopmental disorders, has been only sparingly deployed in studies of Rett syndrome. A number of studies have examined the auditory brainstem response (ABR) to very basic stimulus trains [10–13]. While these studies have revealed that early auditory processing is indeed different from healthy control participants, it is notable that these differences were often relatively subtle. To our knowledge, less than a handful of prior studies have examined higher-level cortical processing of auditory information using ERPs [13–16] and these have yielded both inconsistent findings and interpretations. For example, in a study of seven girls with Rett (aged 10–22), Bader and colleagues reported that although the auditory N1-P2 component complex is *sometimes* delayed in Rett (2/7 patients) its normal topographic distribution and the presence of a significantly different response for standard versus deviant sounds (in an auditory oddball task) signified retained higher hearing functions and the ability to discriminate between novel and non-novel stimuli. On the other hand, in a study by Stauder and colleagues in 2006, where a similar oddball design with simple tone-pip stimuli was employed, the authors reported “reduced ERP differences between task conditions” in patients with Rett, as well as a failure to demonstrate typical developmental changes seen in neurotypical controls in the basic cortical auditory evoked response. They found that participants with Rett had longer ERP latencies and smaller ERP amplitudes than control participants, confirming basic auditory processing deficits. However, and we believe this to be a key observation, despite clear anomalies in the ERP waveforms, it was nonetheless the case that their Rett patients showed relatively robust cortical responses to simple auditory stimuli, suggesting some degree of preservation of early auditory cortical function.

Clearly, much remains to be understood in terms of this residual auditory functioning, and there is a clear need for experiments designed to test more than the initial cortical representation of simple tone pips. Here, we set out to interrogate automatic auditory deviance detection in Rett patients using the mismatch negativity (MMN) component of the ERP as our primary dependent measure. The MMN response is typically evoked by introducing an occasional change (a deviant) to a regularly repeating sequence (termed the standard) of auditory inputs. For example, one might play a series of tones of a given pitch and occasionally introduce a tone of a different pitch—the pitch change will elicit an MMN. Other features of the auditory deviant can be manipulated, such as its duration (a longer tone than in the regular sequence), its loudness, its location, and so on [17–20]. Importantly, the MMN is tightly linked to perceptual capacity, such that the size and latency of the MMN is strongly associated with behavioral discrimination accuracy and speed [21, 22]. In this way, one can use the MMN to assess the integrity of a host of auditory processing functions [23]. Crucially, the MMN, while tightly linked to perception and discrimination, reflects pre-attentive auditory processing [24], and so it can be recorded perfectly well from participants passively exposed to stimulation, often while they are engaged in other activities such as watching a movie or reading a book [25, 26]. This makes it an ideal assay of the integrity of auditory cortical functioning in populations where overt behavioral responses are difficult to ascertain and quantify.

Here, we set out to determine whether children with Rett syndrome would produce an MMN to rare auditory pitch changes, which would indicate intact basic pre-attentive processing for fundamental features of the acoustic signal. On the other hand, attenuations, delays, or frank absence of the MMN would indicate dysfunction and the extent thereof. Establishing baseline measures of MMN integrity in Rett may pave the way to new objective biomarkers of sensory-cortical dysfunction in Rett and provide objective measures against which the efficacy of new therapeutic approaches can be measured.

## Methods

### Participants

Fourteen female patients (mean age 12.41; range 3.9–20.6) with a diagnosis of Rett syndrome participated in this study. They were recruited during clinical visits to the Rett Center at the Children’s Hospital of Montefiore Medical Center in the Bronx, New York. Diagnosis was based on current diagnostic criteria [27] and was confirmed clinically by a medical doctor specializing in this population (A.D.) as well as via genetic testing. Symptom severity was assessed for each patient using the Rett Syndrome Severity Scale (RSSS), as modified by Kaufmann and colleagues [28]. This clinician-rated scale represents an aggregate measure of the severity of clinical symptoms, including motor function, seizures, respiratory irregularities, ambulation, scoliosis, and speech. Each item is scored from 0 (absent/normal) to 3 (severe). Composite scores in the 0–7 range correspond to a mild symptom phenotype, from 8 to 14 to a moderate symptom phenotype, and from 15 to 21 to severe features. Most participants in this study were in the moderate to severe range, and more than half (8 of 14) were not ambulatory. Clinical characteristics of the Rett group are summarized in Table 1. All of the patients were on some form of medication (summarized in Table 2).

**Table 1:** Clinical demographics

Rett participant	Age (years)	Severity score (RSSS)	Epilepsy	Ambulatory	Language
1	3.9	7	No	No	None
2	13.4	14	Yes	No	None
3	8.6	14	Yes	Yes	None
4	16.3	19	Yes	No	None
5	13	15	Yes	No	None
6	13.9	10	Yes	Yes	None
7	5.1	13	No	Yes	None
8	20.6	13	Yes	No	None
9	20.1	6	No	Yes	None
10	16.9	12	Yes	Yes	None
11	6.9	14	No	No	None
12	13.8	16	Yes	No	None
13	10.1	12	No	Yes	None
14	11.1	16	No	No	None

**Table 2:** Medications

Drug class	Benzodiazepine	Valproic acid	Anticonvulsant	SSRI	Other (GI, kidney, asthma)
Rett patients (*N*)	7	9	9	6	11

The patients diagnosed with Rett were compared to a control group of 22 age-matched female participants (mean age 12.49; range 4.3–21.1). Control participants were all typically developing with no familial history of Rett syndrome and no current or lifetime history of psychiatric or neurological disorders.

This study was approved by the institutional review board of The Albert Einstein College of Medicine (Protocol Reference Number #2011-447). Written informed consent was obtained from parents or legal guardians, where possible assent from the patient was also ascertained, and all aspects of the research conformed to the tenets of the Declaration of Helsinki.

### Experimental design (procedures and stimuli)

Participants sat in a darkened sound-attenuated electrically shielded booth (Industrial Acoustics Company, Bronx, NY), either alone (in a chair or wheelchair) or on a parent’s lap, while watching a movie of their choosing on a laptop (*Dell* Latitude E640), with the volume turned off. Auditory stimuli were presented using a pair of speakers (Bose Companion 2 Series II, Multimedia Speaker System) placed behind the laptop. An oddball paradigm was employed whereby standard and deviant auditory stimuli were presented randomly with a likelihood of 0.85 to 0.15, respectively. Auditory stimuli consisted of pure tones at two different frequencies, 503 Hz for the standards and 996 Hz for the deviants. Both tones had a duration of 100 ms, a rise and fall time of 10 ms, and an intensity of 75 dB SPL. Participants ignored the sounds and watched a silent movie. Each block contained 140 stimuli, presented every 900 ms, and the Rett group completed an average of 9.3 blocks (range 7–11) while the control group completed an average of 10.0 blocks (range 9–11).

### EEG recordings

Continuous EEG data were recorded using a Biosemi ActiveTwo 64 electrode array, analog-to-digital converter, and fiber-optic pass-through to a dedicated acquisition computer (digitized at 512 Hz; DC-to-150 Hz pass-band). With the Biosemi system, every electrode or combination of electrodes can be assigned as a reference, which is done purely in software after acquisition. Biosemi replaces the ground electrodes that are used in conventional systems with two separate electrodes: common mode sense and driven right leg passive electrode. These two electrodes create a feedback loop, thus rendering them as references. For more information on the Biosemi system conventions, please visit the website (http://www.biosemi.com/).

### Data processing

All EEG processing and analyses were performed in MATLAB (the MathWorks, Natick, MA, USA) using custom scripts and the FieldTrip Toolbox [41]. Following recording, the continuous EEG was segmented into epochs of 600 ms in length, from −150 to +450 ms. Artifact rejection and channel interpolation procedures were as follows. Bad channels were determined using field trip functions. First, all epochs were arranged by stimulus type and concatenated into two files: one for standards and one for deviants. These were broken into five segments each (of ~93 and 15 s duration, respectively), and bad channels were identified for interpolation as follows: Channels with voltage standard deviation twice that of at least 2 of its 4 nearest neighbors were determined to be overly noisy and marked for interpolation, and channels with voltage standard deviation smaller than one third of the standard deviation of at least 2 out its 4 nearest neighbors were deemed to have too little signal and marked for interpolation. This had to be the case for at least two of the five segments. Prior to interpolation, the data were high pass filtered at 2.15 Hz and a fourth-order filter of 50–95 Hz was applied. The whole concatenated time series for that channel was then interpolated (not just the bad segment) on the basis of all good electrodes within a 4.25-cm radius. Following interpolation, a low pass filter of 30 Hz was applied to all of the data. Prior to averaging the data, if a channel exceeded ±100 μV in a given trial, that trial was removed. This procedure resulted in an imbalance in the number of trials between the two groups. To ensure both groups had equal trial number representation in any further analyses, the proportion of trials from the Rett group versus the control group for each condition was computed at the group level. This fraction was then used as the criterion for the number of trials that would be randomly sub-sampled from the individual control participant data (see Table 3). Epochs were baselined −100 to stimulus onset (0 ms) and then averaged as a function of stimulus condition to yield the auditory evoked potential (AEP) to the standard and to the deviant. These AEPs were then re-referenced to electrode site TP7, which sits on the left hemiscalp slightly above the mastoid. Table 4 shows the number of channels interpolated and the percentage of rejected trials per condition for each of the groups.

**Table 3:** Trial numbers included in the analysis

Trial numbers (*μ* ± *σ*2)	Standard		Oddball	
	Control	Rett	Control	Rett
Original	776 ± 163	489 ± 213	134 ± 32	86 ± 38
*t* test	*p* < 0.0001		*p* < 0.001	
Equalized	485 ± 103	489 ± 213	87 ± 20	86 ± 38
*t* test	*p* = 0.95		*p* = 0.87

**Table 4:** Channel interpolation and percent rejected trials

	Control		Rett		Between groups
	Mean	SD	Mean	SD	*t* test
Number of interpolated channels	3.95	0.63	9.37	1.37	*p* = 0.0043
Percentage of rejected trials—standard	1.45	0.80	15.66	3.10	*p* < 0.001
Percentage of rejected trials—oddball	1.48	1.24	15.28	5.49	*p* < 0.0001

### Derivation of the MMN response

Individual participant data were analyzed for the presence of the MMN by comparing the respective AEPs to the standard and deviant tones. The MMN was expected to onset between 100 and 200 ms post-deviance onset and to be of maximal amplitude over fronto-central scalp sites consistent with prior work and the geometric projection of generators along the surface of the sylvian fissure in the vicinity of Heschl’s gyrus [29]. Visual inspection of the data revealed that for the healthy control group, the MMN (i.e., the difference wave obtained when the ERP to the oddball is subtracted from the ERP to the standard) showed a maximal difference at ~115 ms. A time window of ±10 ms was therefore defined centered at this point of maximal difference and used to extract average amplitudes from the individual participant data for further statistical analysis.

### Exploratory statistical cluster plots

Limiting the analysis to a set of discrete component peaks at electrode sites where the components are maximal represents a highly conservative approach to the analysis of high-density ERP data and raises the likelihood of missed effects (type II errors). Therefore, an exploratory (post hoc) analysis testing the entire data matrix for possible effects was also conducted as a means of fully exploring the richness of our dataset and as a hypothesis-generating tool for future research. To do so, statistical cluster plots (SCPs) were derived by calculating point-wise, paired, two-tailed *t* tests between the AEP generated at each time period for each of the two experimental conditions, for each group, across all scalp sites. This allows for the visualization of any and all significant comparisons between AEPs to the standards and deviants. In order for these AEP comparisons to be considered statistically significant using this clustering approach, the alpha criterion for significance (*p* < 0.05) must be attained for 11 consecutive data points (>20 ms) and for three neighboring electrodes. The rationale for this method is that type I errors are very unlikely to occur simultaneously at adjacent electrodes and equally unlikely to endure for several consecutive time points (i.e., in clusters), even accounting for auto-correlation [30]. The results of the running *t* tests for the 64-electrode array for each AEP comparison are displayed as intensity plots with three major axes to efficiently summarize and facilitate the comparison of the multiple datasets comprising this study. The *x*-axis represents time (post-stimulus onset), the *y*-axis represents electrode (with electrode sites that are adjacent on the scalp appearing next to each other on the plot, also arranged in major groups around the head), and the *z*-axis represents the *t* test result (indicated by a color value from red to blue) at each data point. Areas represented in green do not meet criteria for statistical significance.

## Results

**Fig. 1:**
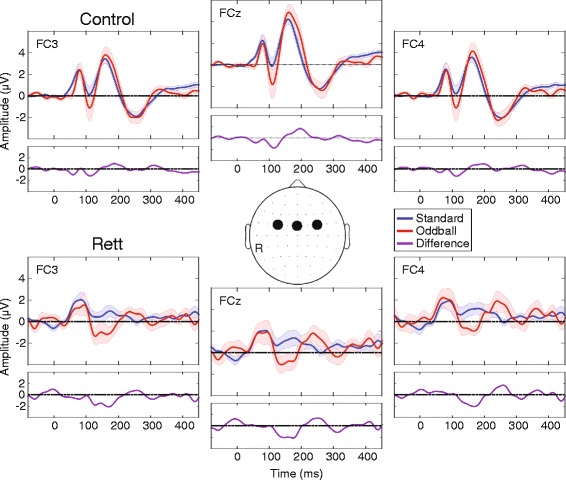
Grand mean waveforms for control (*top*) and patient (*bottom*) groups for electrode sites over fronto-central scalp. The control group shows typical auditory evoked potentials (AEPs), whereas in the Rett group, there is a substantially broader initial cortical response to both the standard and the deviant (the P1; *blue* and *red traces*, respectively), and dramatic attenuation of the N1-P2 complex that is particularly pronounced for the standard. Nevertheless, in the Rett group as in the control group, the oddball stimulus elicited a larger AEP in the N1-P2 timeframe. This is consistent with the MMN response (see *purple trace* for standard minus oddball difference wave) and suggests that automatic change detection occurred. Notably, the MMN appears delayed and of longer duration in the Rett group. *Red* and *blue opaque shading* around the waveform plots reflect standard errors

Event-related potentials over fronto-central scalp show the expected auditory evoked potential in the control group, with an MMN in response to the oddball versus standard condition peaking at 115-ms post-stimulus (Fig. 1). A 2 × 2 mixed repeated measures analysis of variance, with group as an independent factor (Rett and control) and condition as a repeated measure (oddball and standard), was performed at the midline frontal scalp-site FCz for the time period of interest described above (mean amplitude over a 10-ms window centered at the MMN peak at 115 ms). This revealed a significant main effect of condition, *F*(1,34) = 4.41, *p* = 0.043, with the AEP amplitude elicited by the oddball stimulus significantly greater than the AEP amplitude elicited by the standard stimulus. There was no significant main effect of group, *F*(1,34) = 0.84, *p* = 0.37, or group × condition interaction, *F*(1,34) = 0.88, *p* = 0.36. Thus, the ANOVA points to the presence of an MMN in the pooled group of participants in this timeframe. Post hoc tests (reported below), however, show that the MMN was only reliably detectable in the control group at this latency. Note here that the control group was substantially larger than the Rett group and likely drove this main effect.

**Fig. 2:**
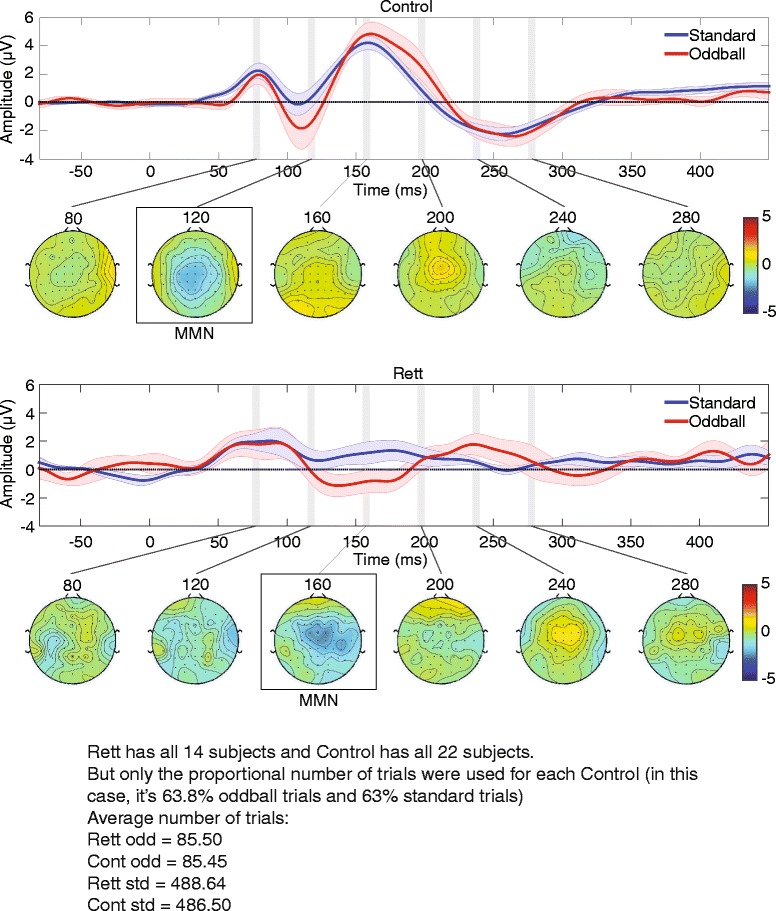
Time course of the MMN (standard minus oddball) for control (*top*) and patient (*bottom*) groups. Scalp topographic maps extracted for 5-ms time windows over prominent peaks at a midline frontal site are depicted (FCz); AEPs to the standard and oddball are included for reference with the time windows shaded in *gray*. The topographic maps further depict the MMN (shaded in *gray*) delay noted in the patient group, with a strong frontal negativity representing this response occurring at 155–160 ms, whereas in the controls, this response is most prominent 115–120 ms. *Red* and *blue opaque shading* around the waveform plots reflect standard errors

**Fig. 3:**
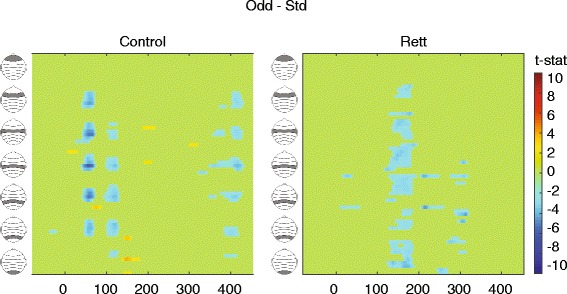
Statistical cluster plots of the MMN: comparing the amplitude of the AEP in response to the standard versus oddball stimulus, for each group. Significant differences are seen in the control group (*left*) over central and fronto-central areas (see also Figs. 1 and 2) at 50–70 ms and 100–120 ms. For the Rett group (*right*), the first wave of significant differences is seen at 130–180 ms, and a second at about 300 ms. Significant *T* values indicate a *t*-stat value of ≥2 for at least 20 consecutive milliseconds and three nearby electrode sites

Figure 2 shows the scalp topography of the standard minus oddball difference wave over several time points. As is typical for the MMN, fronto-centrally focused scalp topographies were observed for both groups. However, the timing of these scalp topographies differed as a function of group and was suggestive of the MMN being later in time for the Rett as compared to the control group (see topographic maps at 160 versus 120 ms). Indeed, the statistical cluster plots provided a fuller picture of the timing and duration of the MMN for each of the groups. These showed that the MMN in the control group had an onset of about 100 ms, whereas in the Rett group, the MMN had a delayed onset of about 130 ms and was of considerably longer duration (Fig. 3; see Fig. 1 as well). We therefore went on to perform a post hoc paired-samples *t* test within each group between the standard and oddball amplitudes in the 10-ms time window centered at 115 ms used in the ANOVA. In this case, there was a significant difference between the two conditions for the controls, *t*(21) = −2.28, *p* = 0.033 (mean deviant = −1.75 (6.09), mean standard = 0.16 (3.41)), but not for the patients *t*(13) = −0.85, *p* = 0.41 (mean oddball = −0.056 (3.27), mean standard = 0.79 (2.84)). To determine if the Rett patients had an intact but delayed MMN, we performed an additional post hoc paired-samples *t* test between the standard and oddball amplitudes where maximal difference was observed. Taking data from a 10-ms time window centered at 152 ms revealed a significant difference in amplitude between the conditions in the Rett group, *t*(13) = −2.72, *p* = 0.017 (mean oddball = −0.91 (2.91), mean standard = 1.13 (2.78)), confirming the presence of a delayed MMN response in Rett syndrome.

## Discussion

It can be particularly challenging to assess the information-processing abilities of individuals with Rett syndrome. As the condition progresses, motor function becomes progressively and stereotypically impaired and there is often a cessation of language production. But while language production is lost in many cases, the degree to which language processing may be preserved remains a question of profound interest to families and clinicians. Here, we probed the integrity of the processing of one of the basic features upon which spoken language is built, auditory frequency contrasts, using non-invasive measures of the cortical auditory processing system. Using the MMN as a probe of discriminative functioning, we found that automatic registration of a large change in pitch appears relatively intact in individuals with Rett syndrome, suggesting that auditory sensory memory and change detection processes are operational in these individuals. At least two aspects of the data, however, call for a degree of moderation when considering this generally positive finding.

The first pertains to our observation that the MMN response was both delayed in its onset and prolonged in its duration. Auditory processing is highly dynamic in nature, and even small delays in the extraction of relevant information could be of significant consequence for the finely tuned process of speech recognition. Thus, the observed delay in the detection of a simple and large frequency change in Rett patients may be of considerable significance when it comes to the processing of more complex and dynamic auditory stimuli such as speech. The protracted duration of the MMN response is more challenging to interpret, since MMN duration has not been previously associated with a specific function that we are aware of. The MMN response itself is thought to represent, at least in part, an updating of auditory sensory memory. Thus, though speculative, one possibility is that the increased duration observed here may represent excessive resource allocation to this process, and this in turn could lead to less efficient updating of sensory memory in Rett syndrome.

The second aspect of the current data that cannot be ignored is that the base AEPs in the Rett group were highly atypical, as can be noted by comparing them with those from the control group in Fig. 1. In fact, it might be considered remarkable that an MMN was elicited at all in the Rett group given the highly atypical morphology and timing of their tone-evoked AEPs to the standard stimuli. While the current study was explicitly designed to assess for the presence of the MMN, the highly anomalous AEPs found here will merit follow-up in future studies. It is also of note that there were two other periods of significant differences between standard and deviant responses observed in post hoc tests of the control dataset, one in the early processing timeframe (circa 50–70 ms) and another in the late processing period centered at about 400 ms (see Fig. 3). Neither of these effects was predicted and would require replication before any clear conclusions could be reached. Nonetheless, their absence in the Rett group is noteworthy, again pointing to highly atypical auditory processing in this group. The early effect in the control group is likely a function of the large pitch difference used to elicit the MMN in this study. This difference may reflect an adaptation effect and would be expected to diminish if smaller pitch differences were assayed. It will certainly be of considerable interest in future work to parametrically vary the extent of pitch difference in Rett syndrome as a means to more fully interrogate the sensitivity of their frequency tuning for change detection. In turn, the late difference at ~400 ms may reflect some form of late processing in higher-order auditory cortices that is not present (or detectable) in the patient cohort.

It is also noteworthy that three studies in independent mouse models of Rett have produced a generally consistent set of findings with regard to basic auditory cortical processing deficits [31–33]. The emergence of new candidate molecular mechanisms of auditory ERP deficits in the Rett mouse model [32] is highly significant from a translational perspective, in that this provides new avenues for possible intervention. It will clearly be critical to develop a much more complete understanding of auditory processing deficits in the patients themselves as this promising line of work goes forward.

While the current work does suggest that at least the early stages of the cortical auditory hierarchy show intact processing capacities for pitch differences, this study represents only a beginning step in the characterization of cortical processing abilities in this population. It is also important to note that the neural measure we used to determine that frequency processing was intact in the auditory cortex of Rett girls, the MMN component of the ERP, does not require explicit engagement on the part of the listener for its evocation [25, 29, 34–36]. That is, it is considered a largely pre-attentive measure of cortical processing and, although in healthy individuals it has been strongly linked to discrimination abilities [21, 22], it cannot with certainty be taken to indicate that the individual can actively manipulate the information in conscious awareness [37]. Nonetheless, the MMN remains a very powerful tool for understanding neurocognitive function in Rett syndrome in that it allows us to determine whether the brain can represent the information units of interest at all. In this way, we can use the MMN as an objective tool to explore basic sensory-perceptual representations of the environment and establish whether the building blocks of cognition are in fact intact. A systematic examination of the auditory processing capabilities of children with Rett syndrome is clearly warranted, and the current work points to the feasibility of this approach.

Recent work in the visual system also points to the utility of similar non-invasive cortical evoked potential measures in this population [38]. In a relatively large sample of females diagnosed with Rett, this research group showed that EEG collected over a matter of a few minutes was enough to characterize the visual evoked potential (VEP) in this population, with significant differences in the VEP morphology seen not only when comparing patients and controls but also within the patient group based on genetic mutation and clinical symptom severity. Also of note, the authors showed similar VEP findings in *Mecp2* heterozygous female mice using an analog paradigm to the one employed in their human sample. Even though the VEP and the AEP/MMN are all obligatory sensory responses, the use of a passive auditory experimental paradigm may have some advantages over VEP studies going forward, in that it imposes lower attention and motor demands on the participant. The fact that the AEP and MMN can be easily elicited without concerns about head position, eye position, or any active engagement with the stimuli, suggests that these measures could ultimately prove more reliable and can be more readily acquired. Further, if a longitudinal study is necessary to track disease progression, a design such as the one reported here may be ideal since patients with a wider variety of clinical severity profiles will be able to complete the study, even in the cases where cognitive and motor skills are subsequently lost.

### Study limitations

Our current cohort of 14 Rett participants is relatively small, and more problematically, the age range covered is quite wide (from approximately 4 to 21 years of age). We know from prior work that the auditory evoked potential and the MMN show significant maturational changes across childhood [39, 40] which will have contributed to increased variance terms herein. Future work should ideally record from a much larger and age-representative sample or limit the cohort to a more delimited age-range. Nonetheless, given the rarity of Rett syndrome, the current study reports data from one of the larger samples tested to date using the ERP methodology. Another limitation pertains to the fact that all the Rett patients were receiving some form of medication (anticonvulsants, benzodiazepines, serotonin reuptake inhibitors, etc.), which may have impacted the electrophysiological responses we recorded here. Recording from medication-free Rett patients, however, is simply not an option. One possible strategy to control for this factor going forward might be to match for medication in another patient group, although given the multiplicity of medications used in this population, this might prove extremely challenging. Also, while we believe there is a highly compelling case in the present data for a delayed onset of the MMN in the patient cohort (based on the post hoc follow-up tests and the SCPs), the planned analysis did not yield a group by condition interaction and therefore one should be cautious at this juncture in generalizing from these data. Lastly, it will be of significant interest to determine whether the presence or absence of the MMN is related to the severity of the symptoms in this population. This, however, would require statistical analysis at the individual participant level, something not afforded by the current dataset where the average number of accepted deviant trials is not sufficient for single-trial analysis.

## Conclusion

It becomes increasingly difficult for clinicians and caretakers to accurately assess the level of preserved auditory functioning in Rett patients due to the decline that is prognostic of the disorder. Non-invasive electrophysiological techniques allow for objective measures of auditory processing without the need for overt behavioral responses. Using these techniques, the current study showed the presence of an MMN response in Rett patients, which suggests preserved abilities to process pitch changes in auditory sensory memory. This work is a beginning step in an effort to comprehensively map the extent of auditory cortical functioning in Rett syndrome. These easily obtained objective brain measures of auditory processing have promise as biomarkers against which future therapeutic efforts can be assayed.

## References

[1] Djukic A (2014). Rett syndrome: recognition of facial expression and its relation to scanning patterns. Pediatr Neurol.

[2] Rose SA (2013). Rett syndrome: an eye-tracking study of attention and recognition memory. Dev Med Child Neurol.

[3] Djukic A (2012). Rett syndrome: basic features of visual processing-a pilot study of eye-tracking. Pediatr Neurol.

[4] Djukic A, McDermott MV (2012). Social preferences in Rett syndrome. Pediatr Neurol.

[5] von Tetzchner S (1996). Vision, cognition and developmental characteristics of girls and women with Rett syndrome. Dev Med Child Neurol.

[6] Baptista PM (2006). Cognitive performance in Rett syndrome girls: a pilot study using eyetracking technology. J Intellect Disabil Res.

[7] Velloso Rde L, de Araujo CA, Schwartzman JS (2009). Concepts of color, shape, size and position in ten children with Rett syndrome. Arq Neuropsiquiatr.

[8] Foxe JJ, Simpson GV (2002). Flow of activation from V1 to frontal cortex in humans. A framework for defining “early” visual processing. Exp Brain Res.

[9] Byiers B, Symons F (2013). The need for unbiased cognitive assessment in Rett syndrome: is eye tracking the answer?. Dev Med Child Neurol.

[10] Pillion JP (2003). Prevalence of hearing loss in Rett syndrome. Dev Med Child Neurol.

[11] Galbraith GC, Philippart M, Stephen LM (1996). Brainstem frequency-following responses in Rett syndrome. Pediatr Neurol.

[12] Pelson RO, Budden SS (1987). Auditory brainstem response findings in Rett syndrome. Brain Dev.

[13] Stach BA (1994). Auditory evoked potentials in Rett syndrome. J Am Acad Audiol.

[14] Bader GG, Witt-Engerstrom I, Hagberg B (1989). Neurophysiological findings in the Rett syndrome, II: visual and auditory brainstem, middle and late evoked responses. Brain Dev.

[15] Peters SU, Gordon RL, Key AP (2015). Induced gamma oscillations differentiate familiar and novel voices in children with MECP2 duplication and Rett syndromes. J Child Neurol.

[16] Stauder JE (2006). The development of visual- and auditory processing in Rett syndrome: an ERP study. Brain Dev.

[17] De Sanctis P (2009). Right hemispheric contributions to fine auditory temporal discriminations: high-density electrical mapping of the duration mismatch negativity (MMN). Front Integr Neurosci.

[18] De Sanctis P (2008). Auditory scene analysis: the interaction of stimulation rate and frequency separation on pre-attentive grouping. Eur J Neurosci.

[19] Butler JS (2011). Common or redundant neural circuits for duration processing across audition and touch. J Neurosci.

[20] Butler JS (2012). Multisensory representation of frequency across audition and touch: high density electrical mapping reveals early sensory-perceptual coupling. J Neurosci.

[21] Sams M (1985). Auditory frequency discrimination and event-related potentials. Electroencephalogr Clin Neurophysiol.

[22] Pakarinen S (2007). Measurement of extensive auditory discrimination profiles using the mismatch negativity (MMN) of the auditory event-related potential (ERP). Clin Neurophysiol.

[23] Naatanen R (2007). The mismatch negativity (MMN) in basic research of central auditory processing: a review. Clin Neurophysiol.

[24] Naatanen R, Jacobsen T, Winkler I (2005). Memory-based or afferent processes in mismatch negativity (MMN): a review of the evidence. Psychophysiology.

[25] Ritter W (2006). Preattentively grouped tones do not elicit MMN with respect to each other. Psychophysiology.

[26] Ritter W (2002). Memory reactivation or reinstatement and the mismatch negativity. Psychophysiology.

[27] Neul JL (2010). Rett syndrome: revised diagnostic criteria and nomenclature. Ann Neurol.

[28] Kaufmann WE (2012). Social impairments in Rett syndrome: characteristics and relationship with clinical severity. J Intellect Disabil Res.

[29] Molholm S (2005). The neural circuitry of pre-attentive auditory change-detection: an fMRI study of pitch and duration mismatch negativity generators. Cereb Cortex.

[30] Guthrie D, Buchwald JS (1991). Significance testing of difference potentials. Psychophysiology.

[31] Goffin D (2012). Rett syndrome mutation MeCP2 T158A disrupts DNA binding, protein stability and ERP responses. Nat Neurosci.

[32] Goffin D (2014). Cellular origins of auditory event-related potential deficits in Rett syndrome. Nat Neurosci.

[33] Liao W (2012). MeCP2+/− mouse model of RTT reproduces auditory phenotypes associated with Rett syndrome and replicate select EEG endophenotypes of autism spectrum disorder. Neurobiol Dis.

[34] Kornilov SA (2014). Attentional but not pre-attentive neural measures of auditory discrimination are atypical in children with developmental language disorder. Dev Neuropsychol.

[35] Leitman DI (2009). Mismatch negativity to tonal contours suggests preattentive perception of prosodic content. Brain Imaging Behav.

[36] Molholm S (2014). Mapping phonemic processing zones along human perisylvian cortex: an electro-corticographic investigation. Brain Struct Funct.

[37] Molholm S, Gomes H, Ritter W (2001). The detection of constancy amidst change in children: a dissociation of preattentive and intentional processing. Psychophysiology.

[38] LeBlanc JJ (2015). Visual evoked potentials detect cortical processing deficits in Rett syndrome. Ann Neurol.

[39] Brandwein AB (2011). The development of audiovisual multisensory integration across childhood and early adolescence: a high-density electrical mapping study. Cereb Cortex.

[40] Gomes H (2001). Spatiotemporal maturation of the central and lateral N1 components to tones. Brain Res Dev Brain Res.

[41] Oostenveld R, Fries P, Maris E, Schoffelen JM. FieldTrip: Open Source Software for Advanced Analysis of MEG, EEG, and Invasive Electrophysiological Data. Computational Intelligence and Neuroscience 2011:1–9. 10.1155/2011/156869 PMC3021840 21253357

